# A unified stochastic modelling framework for the spread of nosocomial infections

**DOI:** 10.1098/rsif.2018.0060

**Published:** 2018-06-13

**Authors:** Martín López-García, Theodore Kypraios

**Affiliations:** 1 School of Mathematics, University of Leeds, LS2 9JT Leeds, UK; 2 School of Mathematical Sciences, University of Nottingham, NG7 2RD Nottingham, UK

**Keywords:** hospital-acquired or nosocomial infections, antibiotic-resistant bacteria, infection control, stochastic model, Markov chain, reproduction number

## Abstract

Over the last years, a number of stochastic models have been proposed for analysing the spread of nosocomial infections in hospital settings. These models often account for a number of factors governing the spread dynamics: spontaneous patient colonization, patient–staff contamination/colonization, environmental contamination, patient cohorting or healthcare workers (HCWs) hand-washing compliance levels. For each model, tailor-designed methods are implemented in order to analyse the dynamics of the nosocomial outbreak, usually by means of studying quantities of interest such as the reproduction number of each agent in the hospital ward, which is usually computed by means of stochastic simulations or deterministic approximations. In this work, we propose a highly versatile stochastic modelling framework that can account for all these factors simultaneously, and which allows one to exactly analyse the reproduction number of each agent at the hospital ward during a nosocomial outbreak. By means of five representative case studies, we show how this unified modelling framework comprehends, as particular cases, many of the existing models in the literature. We implement various numerical studies via which we (i) highlight the importance of maintaining high hand-hygiene compliance levels by HCWs, (ii) support infection control strategies including to improve environmental cleaning during an outbreak and (iii) show the potential of some HCWs to act as super-spreaders during nosocomial outbreaks.

## 1. Introduction

The risk of acquiring nosocomial infections is a recognized problem in healthcare facilities worldwide [1]. It has been estimated that nosocomial infections affect more than 4 million patients in Europe each year, leading to €7 billion of direct medical costs [2]. Moreover, the emergence and spread of antibiotic resistance among these pathogens has posed a second major problem worldwide, stressing the need for understanding their transmission routes in healthcare facilities, and to identify the most effective infection control strategies in these settings [3]. A paradigmatic example of an antibiotic-resistant nosocomial pathogen is bacteria *Staphylococcus aureus* (SA), which is a normal inhabitant of the skin and mucosal surfaces, but can cause different infections when it flourishes in other areas (e.g. soft tissue, bloodstream or lung infections). SA resistance against penicillin-like antibiotics arose a few years after the introduction of penicillin. Moreover, methicillin-resistant SA (MRSA) strains were reported in Europe after only 2 years of the introduction of methicillin in 1959 [4]. Currently, new strains of MRSA have been reported which are also resistant to vancomycin [4].

Healthcare environments such as hospitals or nursing homes are ideal settings for the spread of multidrug-resistant bacteria (MDRB), due to, among other reasons, opportunities for bacteria to enter into the bloodstream or infect open wounds, the presence of immunocompromised and aged individuals, and the high exposure levels to antibiotics [5,6]. The precise mode of transmission is uncertain for many nosocomial pathogens, but usually both exogenous (e.g. cross-colonization) and endogenous (e.g. selective pressure of antibiotics) routes are considered as feasible for these pathogens [3]. While for some nosocomial infections most of the transmission is considered to occur via HCW–patient contact routes [7], there is increasing recognition in the literature of the potential role played by environmental contamination and airborne spread [8–10].

Infection control strategies usually implemented in hospital settings include, among others, hand disinfection procedures, environmental cleaning, active screening for colonization among patients and isolation of colonized individuals, managing staffing levels, antibiotic prescription and decolonization procedures, or patient cohorting [11]. However, control procedures followed in healthcare facilities worldwide usually amount to combinations of the individual interventions listed above, so that the efficacy of each individual strategy is hard to measure. On the other hand, the application of classical epidemiology procedures for addressing this individual efficacy is often not feasible due to financial and ethical restrictions [4,12]. Thus, mathematical modelling is one of the best tools available for understanding the role played by different factors on the emergence and spread of these pathogens and their antibiotic resistance, while measuring the impact of individual interventions [8,13].

A wide range of deterministic and stochastic mathematical models for the spread of nosocomial pathogens have been developed during the last years [2]. Although deterministic models were originally proposed for capturing the main infection dynamics in single wards and hospitals, modelling efforts were soon redirected towards the stochastic perspective due to the small and highly heterogeneous populations usually present in these settings. From a stochastic perspective, most of the models proposed in the literature are based on Markov processes, where it is assumed that inter-event times are exponentially distributed. This simplifying assumption is usually crucial for analytically and computationally treating the processes under study; we refer the reader to Pelupessy *et al.* [3] for a discussion on the advantages of stochastic (in particular, Markovian) approaches, and to van Kleef *et al.* [2] and Assab *et al.* [14] for systematic reviews in this field. Stochastic models in this area can be classified as *compartment-based*, where the population of individuals is classified in groups according to their state against the disease, and wide homogeneities are assumed among the members within the same group, or *agent-based*, where one keeps track of the state of each individual within the population throughout time, allowing one to model heterogeneities at the individual level [8]. Agent-based models can incorporate heterogeneity in, for example, transmission risk profiles of specific patients or HCWs [15], but are usually restricted to the implementation of stochastic simulations in small wards, and are computationally constrained [2].

When constructing and studying these stochastic models, efforts have been focused, and tailor-designed analytical and numerical methods have been implemented, in order to analyse the dynamics of the nosocomial outbreak when accounting for spontaneous colonization of patients, patient-to-staff and staff-to-patient contamination/colonization, environmental contamination, patient cohorting, room configuration of the hospital ward, staff hand-washing compliance levels, the presence of different types of HCWs or specific staff–patient contact network structures. This analysis is usually carried out by means of studying summary statistics directly related to the nosocomial outbreak, such as the reproduction number of each particular agent (e.g. of a colonized patient or a contaminated healthcare worker) in the hospital ward. This is usually computed in an approximative fashion, for example by means of stochastic simulations or in terms of deterministic approximations [16]. On the other hand, the limitations of analysing these processes by simulation approaches, and the convenience of following exact procedures instead when dealing with small populations (such as those usually involved in nosocomial outbreaks), have been highlighted in [17].

In this work, we propose a versatile stochastic modelling framework that can simultaneously account for all the factors listed above, and which allows in §2 for the exact and analytical study of the reproduction number of each agent at the hospital ward during the nosocomial outbreak. We make use of five representative case studies in §3, regarding both hypothetical and real nosocomial outbreaks at hospital wards, to show how this unified modelling framework comprehend, as particular cases, many of the existing models in the field. We conduct several numerical studies and our results in §3 highlight the importance of maintaining high hand-hygiene compliance levels by healthcare workers, support control strategies including to improve environmental cleaning during nosocomial outbreaks and show the potential of some healthcare workers to act as *super-spreaders* during these outbreaks.

## 2. A unified stochastic modelling framework

In this section, we propose the unified stochastic modelling framework for the spread of nosocomial infections, where agents represented in the model can be of different *type* (patients, HCWs, surfaces, patients located in different rooms, etc.). This general framework, which is constructed in terms of a continuous-time Markov chain, allows one to follow an exact and analytical approach for computing the reproduction number of each different *agent* playing a role in the infection spread, which measures the number of *infections* directly caused by this agent until the agent stops spreading the nosocomial pathogen. We also show how this reproduction number can be exactly analysed while deciphering among which individuals this agent is spreading the disease, so that this becomes a quantitative measure of the infectiousness of a given *agent* among individuals of different *type*. This then becomes a useful tool when analysing the role played by different routes of infection during a nosocomial outbreak in a given hospital ward, as shown in numerical results in §3.

### 2.1. The model

**Figure 1.**
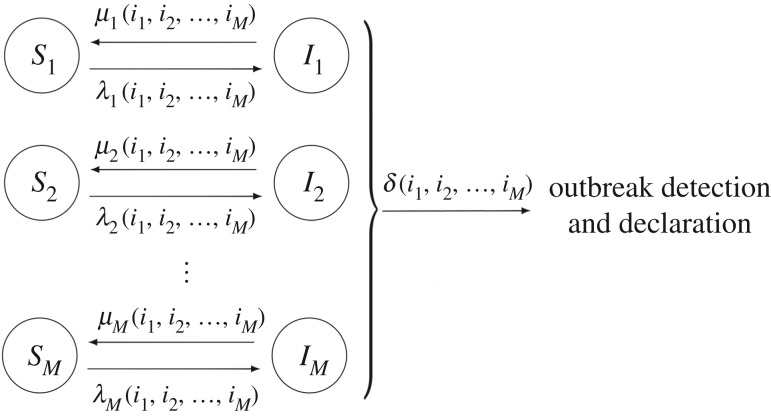
Diagram representing the epidemic dynamics among *M* different compartmental levels.

We consider model depicted in figure 1, which amounts to a stochastic SIS epidemic model with multiple *compartmental levels*. In case studies 1–5 in §3, this modelling framework is used to represent the spread of nosocomial infections, such as MDRB, within a hospital ward, where the meaning of a compartmental level depends on the particular case study, showing the versatility and flexibility of this unified framework.

We consider the stochastic process 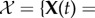 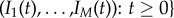, where *I*_*j*_(*t*) amounts to the number of infectives in compartmental level *j* at time *t*≥0. We assume that the number of individuals at each compartmental level remains constant throughout time, which is directly related to standard assumptions when modelling nosocomial infections (see §3). This means that the number *S*_*j*_(*t*) of susceptibles in compartmental level *j* at time *t* is given by *S*_*j*_(*t*) = *N*_*j*_ − *I*_*j*_(*t*) for all *t*≥0. Process 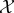 evolves among states in 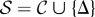, where
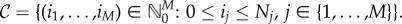
State (*i*_1_, …, *i*_*M*_) represents the presence of *i*_*j*_ infected individuals at compartmental levels 1 ≤ *j* ≤ *M*, while the final state Δ represents the detection and declaration of the outbreak in the hospital ward. In particular, process 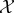 transits among states in 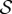 according to the following transitions:

— *Removal* **at compartmental level *j***: 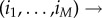 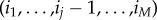, occurring at rate *μ*_*j*_(*i*_1_, …,*i*_*M*_);
— *Infection* **at compartmental level *j***: 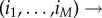 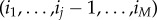, occurring at rate *λ*_*j*_(*i*_1_, …,*i*_*M*_);
— **Detection and declaration of the outbreak**: 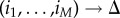, occurring at rate *δ*(*i*_1_, …,*i*_*M*_).

This unified model has been developed to account for patients, different types of HCWs and/or surfaces involved in a nosocomial outbreak in a hospital ward. The generality of functions *λ*_*j*_(*i*_1_, …,*i*_*M*_), *μ*_*j*_(*i*_1_, …,*i*_*M*_) and *δ*(*i*_1_, …,*i*_*M*_) allows for incorporating into the model a wide range of factors having an impact on the nosocomial spread dynamics. This means that the particular meaning of each compartmental level 1 ≤ *j* ≤ *M*, as well as of each event (infections and removals represented by arrows in figure 1) depends on the particular hospital ward and pathogen under analysis; see §3 where compartmental levels 1 ≤ *j* ≤ *M* can represent colonized/non-colonized patients, contaminated/non-contaminated HCWs, volunteers and surfaces, or can be related to the specific spatial configuration of the hospital ward under analysis, or the particular staff–patient contact network (e.g. representing patient cohorting).

Outbreak detection and declaration rate *δ*(*i*_1_, …,*i*_*M*_) allows one to analyse situations where a nosocomial pathogen is introduced for the first time in a given hospital ward (e.g. by admission of a colonized patient), starting an outbreak, and the spread dynamics are analysed until the presence of this pathogen is detected by HCWs. By conveniently specifying the function rate *δ*(*i*_1_, …,*i*_*M*_), different hospital surveillance policies (e.g. detection by the first individual showing symptoms, by random screening of patients within the ward, or by systematic screening upon patient admission) can be considered. However, as illustrated in §3, scenarios where the interest is not in the spread dynamics until detection, but in the long-term infection dynamics of the pathogen (e.g. endemic situations) and in assessing the infectiousness of each agent within this ward, can be analysed by setting *δ*(*i*_1_, …,*i*_*M*_) = 0. We note that setting *δ*(*i*_1_, …,*i*_*M*_) = 0 means deleting the final state Δ in figure 1, so that the infection dynamics during the nosocomial outbreak would amount to the stochastic movement of individuals, throughout time, between the susceptible and infective compartments at the different compartmental levels in figure 1; see case studies 2–5.

In subsection 2.1, and for a given initial state (*I*_1_(0), …, *I*_*M*_(0)) = (*i*_1_, …,*i*_*M*_), we analyse the *exact reproduction number* for an infective individual in compartmental level *j*: the number of infections (understood in a broad sense, see §3) directly caused by this individual until he/she is removed or until the outbreak is detected, *R*^(*j*)^_(*i*_1___, … ,*i*_*M*___)_ [18–20]. Since an infective individual at compartmental level *j* can infect individuals at compartmental levels 1 ≤ *k* ≤ *M*, one can split 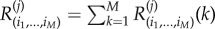, where *R*^(*j*)^_(*i*_1___, … ,*i*_*M*___)_(*k*) is the number of infections directly caused by an infective individual at compartmental level *j*, among individuals at compartmental level *k*. In this way, random variables *R*^(*j*)^_(*i*_1___, … ,*i*_*M*___)_(*k*), for 1 ≤ *j*, *k* ≤ *M*, allow one to assess the role played by the different potential routes of infection during a nosocomial outbreak in a hospital ward, in our numerical results in §3. We note that the global variable *R*^(*j*)^_(*i*_1___, … ,*i*_*M*___)_ measures the infectiousness of an infective individual in compartmental level *j*, until this individual stops spreading the infection (he/she is removed) or until the outbreak is detected and declared (so that control strategies such as antibiotic prescription, isolation of infected individuals, patient cohorting or environmental cleaning, can be implemented, impacting on the infection spread dynamics). These summary statistics can be studied from the solution of systems of linear equations, by implementing first-step arguments. In the electronic supplementary material, we explain the corresponding algorithmic procedures designed for solving these systems in a matrix-oriented fashion.

### 2.2. Reproduction number for an individual at compartmental level *j*, among individuals at compartmental level *k*

For a given compartmental level *j* and a given initial state (*i*_1_, …,*i*_*M*_), we can define the random variable *R*^(*j*)^_(*i*_1___, … ,__*i*_*M*___)_, which amounts to the total number of infections directly caused by a marked infective individual at compartmental level *j* until he/she is removed, or until the outbreak is declared. We note that since quantity *R*^(*j*)^_(*i*_1___, … ,__*i*_*M*___)_ refers to an infective individual at compartmental level *j*, it is only properly defined for initial states (*i*_1_, …,*i*_*M*_) with *i*_*j*_ > 0. In case studies 1–5 in §3, we focus on initial states of the form
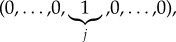
representing that the infective individual under study is the one at compartmental level *j* starting the outbreak. For this initial state, the mean value *E*[*R*^(*j*)^_(0, … ,0,1,0, … ,0)_] directly relates to the *basic reproduction number* (measuring the average number of individuals this individual directly infects until he/she is removed—or, in this case, until the outbreak is detected—for an initially fully susceptible population).

We note that *R*^(*j*)^_(*i*_1___, … ,__*i*_*M*___)_ is in fact the sum of several contributions,
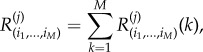
where *R*^(*j*)^_(*i*_1___, … ,__*i*_*M*___)_(*k*) represents the number of infections caused, by this individual who is at compartmental level *j*, only *among* individuals at compartmental level *k*. The analysis of each variable *R*^(*j*)^_(*i*_1___, … ,__*i*_*M*___)_(*k*) helps to measure not only how infectious an individual that belongs to compartmental level *j* is, but also how much of a risk he/she is for individuals at a given compartmental level *k*. This allows us in §3 to explore the role played by the different potential transmission routes during a nosocomial outbreak.

The probability distribution of each random variable *R*^(*j*)^_(*i*_1___, … ,__*i*_*M*___)_(*k*) is given in terms of probabilities
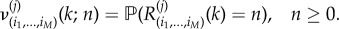
Since these probabilities refer to a particular infected individual, it is necessary to specify the contribution that each infective individual has in the global infection rates *λ*_*j*_(*i*_1_, …,*i*_*M*_), as well as the rate at which this particular individual is removed. Thus, we analyse quantities *R*^(*j*)^_(*i*_1___, … ,__*i*_*M*___)_(*k*) and *R*^(*j*)^_(*i*_1___, … ,__*i*_*M*___)_ for the following family of infection and removal rates:
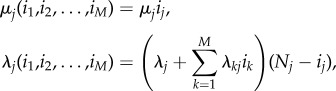
for 1 ≤ *j* ≤ *M*, and any outbreak detection and declaration rate *δ*(*i*_1_, …,*i*_*M*_). This specification of rates is based on the following general assumptions:

— Each infective individual at compartmental level *j* is removed independently at rate *μ*_*j*_;
— Each susceptible individual at compartmental level *j* can be infected due to an external source of infection, with rate *λ*_*j*_, or by an infective individual at compartmental level *k*, with rate *λ*_*kj*_.

We note that these functions have been defined in this way so that they can be used in case studies 1–5 for the spread of nosocomial pathogens in hospital wards, where events related to rates *μ*_*j*_, *λ*_*j*_ and *λ*_*kj*_ have specific meanings in each case study in §3, according to different scenarios and hypotheses considered in [15,19,21–23].

We follow here a first-step argument conditioning on the next event to occur in the process. In particular, for the initial state **i** = (*i*_1_, …, *i*_*M*_), we have2.1
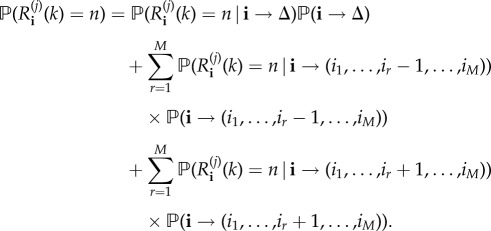
Notation 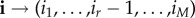 represents the event that, if the process is at state **i** at present time, the next event that occurs in the process is the transition to state (*i*_1_, …, *i*_*r*_ − 1, …, *i*_*M*_) (i.e. a removal occurs at compartmental level *r*). The equation above, if we use notation
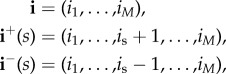
leads to the system of equations:


2.2
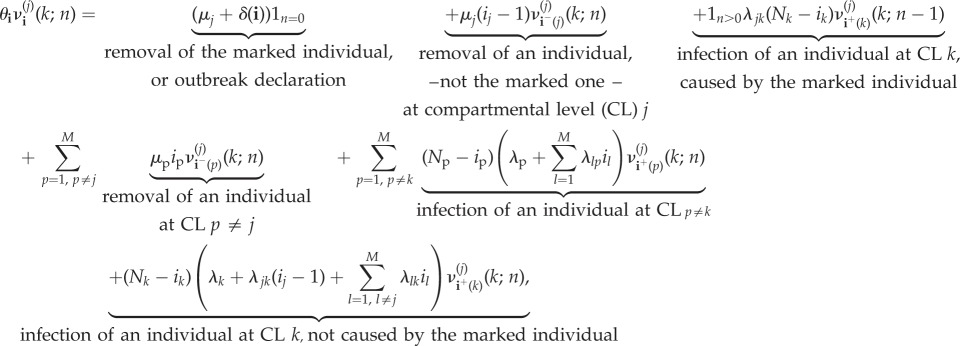


for *n*≥0 and 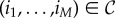, with *i*_*j*_ > 0. 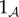 above is a function equal to 1 if 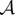 is satisfied, and 0 otherwise, and
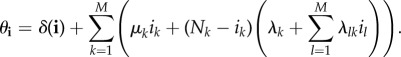
We note that equation (2.2) is obtained by following arguments in equation (2.1), and conditioning on the next event that can potentially occur in the process. For example, let us assume that process is at state **i** = (*i*_1_, …,*i*_*M*_) at present time, and we are computing probability 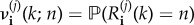, which relates to the reproduction number *R*^(*j*)^_**i**_(*k*) for a marked infective individual at compartmental level *j*, among individuals at compartmental level *k*. A potential event which can occur is the recovery of an individual—different to the marked one—at compartmental level *j*, which by the theory of Markov processes occurs with probability *μ*_*j*_(*i*_*j*_ − 1)/*θ*_(*i*_1___, … ,__*i*_*M*___)_, moving the process to the new state **i**^−^(*j*) = (*i*_1_, …, *i*_*j*_ − 1, …,*i*_*M*_). This leads to the addend *μ*_*j*_(*i*_*j*_ − 1)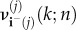 in equation (2.2), and similar arguments can be applied for the rest of potential possible events that can occur. Finally, we point out that the system of equations given by equation (2.2) can be represented in matrix form, and solved by starting with *n* = 0, and then sequentially solving the system of equations for any value *n*≥1 by using previously computed probabilities for *n* − 1, in an iterative fashion; see the electronic supplementary material.

It is clear that, since
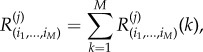
we can also focus on computing probabilities
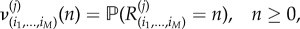
for any initial state 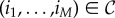 with *i*_*j*_ > 0. Probabilities *ν*^(*j*)^_(*i*_1___, … ,*i*_*M*__)__(*n*) satisfy
2.3
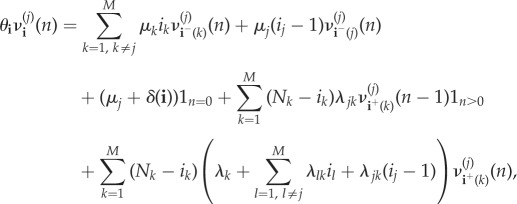
for *n*≥0 and for any 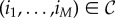, with *i*_*j*_ > 0. This system is expressed in matrix form, and solved in an iterative fashion, in the electronic supplementary material.

## 3. Case studies

In this section, we focus on five different representative existing models in the literature for the spread of nosocomial infections. Our aim is to show how these models can be seen as particular cases of the unified stochastic modelling framework presented in §2, so that the methodology in subsection 2.1 can be directly applied, and the infectiousness of each agent in the hospital ward can appropriately be quantified. In particular, case studies 1–5 can be represented into our framework by specifying the number *M* of compartmental levels and their meaning, as well as the meaning of the infection and removal events occurring at each compartmental level, and the specifications of rates *μ*_*j*_, *λ*_*j*_, *λ*_*jk*_ and *δ*(*i*_1_, …, *i*_*M*_). These rates are general enough in §2 in order to account for all hypotheses usually considered when modelling nosocomial infections (such as those considered in [15,19,21–23] related to case studies 1–5), and also allow one to consider different hospital surveillance policies for outbreak detection and declaration [24,25]. A summary of these rates for each case study studied in this section can be found in the electronic supplementary material, Table S6.

### 3.1. Modelling spread among patients and healthcare workers

We focus here on the model by Artalejo [21], for a nosocomial outbreak in a hospital ward with *N*_p_ patients and *N*_HCW_ HCWs. Patients can be colonized or non-colonized at any given time, and are discharged at rate *μ*, regardless of their colonization status. HCWs can have their hands contaminated or uncontaminated, and they wash their hands at rate *μ*′. Each colonized patient *contaminates* (the hands of) each uncontaminated HCW at rate *β*′, while each contaminated HCW colonizes each non-colonized patient at rate *β*. Admission of new patients occurs immediately after discharge, and newly admitted patients can be colonized with probability *σ*. It is assumed in [21] that each colonized patient is detected at rate *γ*, which can be incorporated here by setting *δ*(*i*_1_, *i*_2_) = *γi*_1_ (i.e. outbreak declaration occurs upon detection of the first colonized patient).

We note that the outbreak detection and declaration rate *δ*(*i*_1_, …, *i*_*M*_) can be set to account for different hypotheses regarding hospital surveillance and screening. By setting *δ*(*i*_1_, *i*_2_) = *γi*_1_ as above, one can represent random screening being in place as the surveillance policy in the hospital ward, where each patient is screened at an average time *γ*^−1^ [24], where this screening policy is identified as one of the most efficient ones for the control of nosocomial outbreaks. We also note that outbreak declaration rate *δ*(*i*_1_, *i*_2_) = *γi*_1_ can also be used to represent the scenario where outbreak is declared after the first colonized patient showing some symptoms, each colonized patient showing symptoms at rate *γ* (e.g. norovirus outbreaks are declared upon detection of suspected cases, consisting of patients showing symptoms such as diarrhoea and vomiting). On the other hand, if a colonized patient is admitted into a hospital ward, and detection occurs by screening upon admission where laboratory results take an average time *δ*^−1^ to arrive, one could represent this by setting *δ*(*i*_1_, *i*_2_) = *δ* and with time *t* = 0 representing the admission of the colonized patient into the ward.

**Figure 2.**
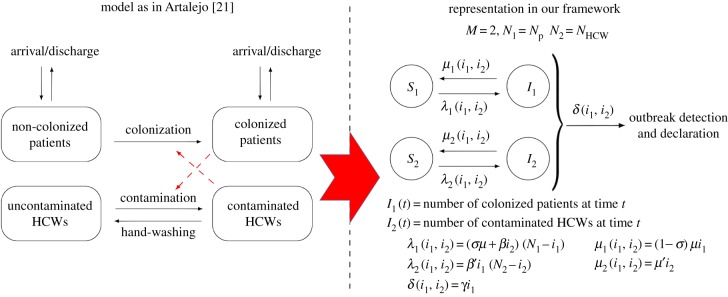
Model by Artalejo [21] and its corresponding representation in our framework. Our representation leads to the same stochastic process to that in [21]. Case study 1. (Online version in colour.)

In figure 2, we show how this model can be represented into our framework, by setting *M* = 2, *N* = *N*_p_ + *N*_HCW_, where compartmental level *j* = 1 amounts to colonized/non-colonized patients and *j* = 2 amounts to uncontaminated/contaminated HCWs. In order to incorporate the hypotheses above, rate functions *λ*_*j*_(*i*_1_, *i*_2_), *μ*_*j*_(*i*_1_, *i*_2_) and *δ*(*i*_1_, *i*_2_) are defined as in figure 2, and summarized in the electronic supplementary material, Table S6. Moreover, summary statistics analysed in §2 have specific meanings in this particular case study, as described in table 1. We note here that an alternative existing approach in the literature, such as the model in [3], is to consider only colonized/non-colonized patients explicitly in the model, where the role played by contaminated HCWs is only implicitly incorporated via a transmission rate *β*. Model in [3] could be represented into our framework by setting *M* = 1 (colonized/non-colonized patients) and appropriately setting rates *μ*_1_(*i*_1_), *λ*_1_(*i*_1_) and *δ*(*i*_1_), which is omitted here for the sake of brevity.

**Table 1.** Meaning of our summary statistics for model in figure 2. Case study 1.

*R*^(1)^_(1,0)_ = *R*^(1)^_(1,0)_(2)	reproduction number of a colonized patient starting the outbreak (among HCWs)
*R*^(2)^_(0,1)_ = *R*^(2)^_(0,1)_(1)	reproduction number of a contaminated HCW starting the outbreak (among patients)

We use here parameter values considered in [21], for the spread of MRSA in an hypothetical intensive care unit, which are reported in the electronic supplementary material, Table S1. When analysing the infectiousness of colonized patients and contaminated HCWs, we can focus on computing the reproduction number of these individuals, as described in §2 (table 1). While the reproduction number can be computed, for a contaminated HCW (*R*^(2)^_(0,1)_), by direct application of equation (2.3), a slight modification needs to be considered when analysing the reproduction number of a colonized patient; that is, when computing probabilities 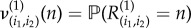. In particular, equation (2.3) for model and rate functions in figure 2 leads to3.1
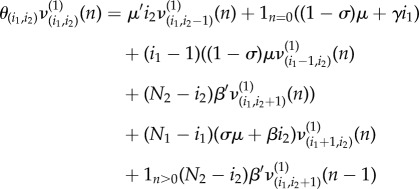
with *θ*_(*i*_1__,*i*_2___)__ = *μ*′*i*_2_ + (1 − *σ*)*μ*(*i*_1_ − 1) + (*N*_1_ − *i*_1_)(*σμ* + *βi*_2_) + (*N*_2_ − *i*_2_)*β*′*i*_1_ + (1 − *σ*)*μ* + *γi*_1_. However, we note that *R*^(1)^_(1,0)_ should amount to the number of infections (i.e. in this case, HCW hands contaminations) directly caused by a given colonized patient starting the outbreak until this patient is discharged or the outbreak is detected, regardless of the newly admitted patient being or not colonized. This means that terms 1_*n*=0_(1 − *σ*)*μ* in equation (3.1) and (1 − *σ*)*μ* in *θ*_(*i*_1___,*i*_2___)_ need to be replaced by 1_*n*=0_*μ* and *μ*, respectively, and the same applies when analysing the reproduction number of a colonized patient in case studies 2–4.

**Figure 3.**
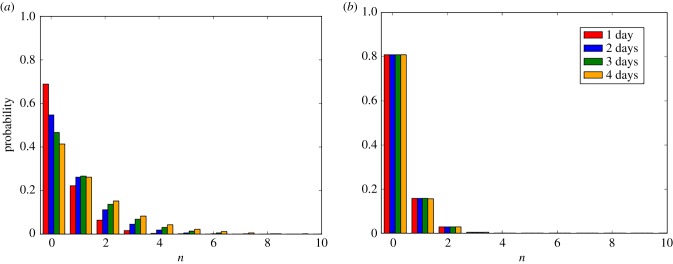
Probability mass functions of the reproduction number of a colonized patient (*R*^(1)^_(1,0)_, *a*) and of a contaminated HCW (*R*^(2)^_(0,1)_, *b*) starting the outbreak. Average detection time of each patient *γ*^−1^ ∈ {1, 2, 3, 4} days. Case study 1.

In figure 3, we plot the probability mass functions of the reproduction number of a colonized patient (*R*^(1)^_(1,0)_) and of a contaminated HCW (*R*^(2)^_(0,1)_) starting the outbreak. While the average outbreak declaration time is crucial for limiting the reproduction number of a colonized patient, this is not the case when looking at the reproduction number of a contaminated HCW. This is related to the fact that the main limiting factor for the infectiousness of a HCW is his/her hand-washing rate, which is something that we explore in more depth in the following case studies.

### 3.2. Considering different healthcare worker types

**Figure 4.**
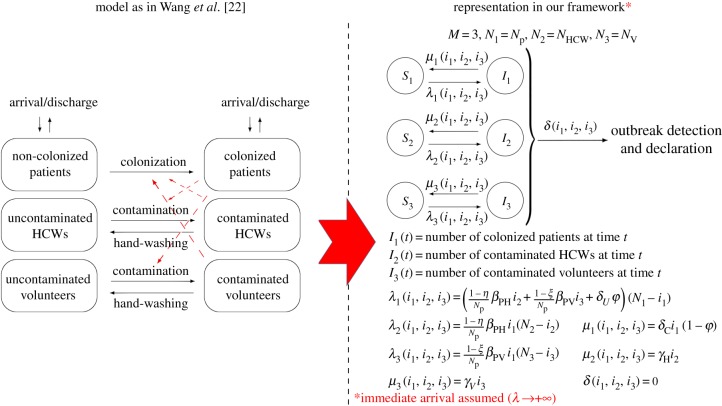
Model by Wang *et al.* [22] and its corresponding representation in our framework. Our representation leads to the same stochastic process to that in [22], when 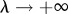. Case study 2. (Online version in colour.)

We focus here on the model by Wang *et al.* [22], which incorporates volunteers working at the hospital ward. They also consider the spread of MRSA in the respiratory intensive care unit (RICU) at Beijing Tongren Hospital, which is formed by *N*_p_ patients, *N*_HCW_ HCWs and *N*_*V*_ volunteers. As assumed in [22], patients are admitted at rate *λ*, who can already be colonized upon admission with probability *φ*, and discharged at rate *δ*_C_ (if colonized) or *δ*_U_ (if non-colonized). HCW–patient transmission rate *β*_PH_(1 − *η*)/*N*_P_ consists of two contributions: the hygienic level *η* ∈ (0, 1) during each HCW–patient contact, which is encoded in a probability (1 − *η*) of transmission per contact, and a contact rate *β*_PH_, and similar comments apply to volunteer–patient transmission rate *β*_PV_(1 − *ξ*)/*N*_P_ (for details, see [22, p. 3] and related equations in [22, appendix]). In figure 4, we depict how this model is represented into our framework, in the asymptotic situation where immediate arrival of patients is assumed after discharge (i.e. 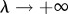), which is a reasonable approximation for hospital wards under high demand [3,23]. Since no detection is considered in [22], where the interest is in the long-term dynamics of the nosocomial spread and in analysing the infectiousness of each individual in the ward, we set *δ*(*i*_1_, …, *i*_*M*_) = 0.

**Figure 5.**
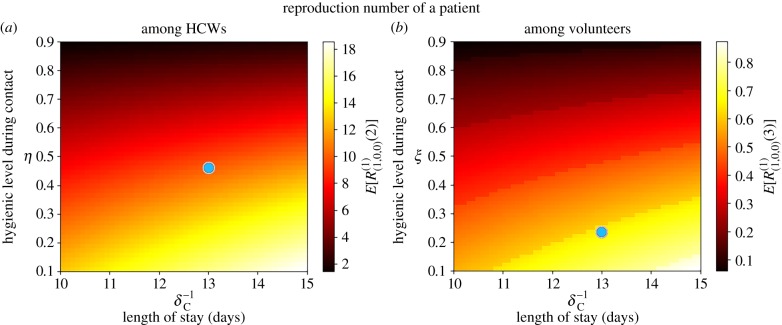
Mean reproduction number of a colonized patient starting the outbreak, among HCWs (*E*[*R*^(1)^_(1,0,0)_(2)], *a*) and volunteers (*E*[*R*^(1)^_(1,0,0)_(3)], *b*), versus *δ*^−1^_C_, *η* and *ξ*. Blue dot corresponds to parameter values (*η*, *ξ*, *δ*^−1^_*C*_) = (0.46, 0.23, 13.0) in electronic supplementary material, Table S2, leading to values *E*[*R*^(1)^_(1,0,0)_(2)] = 10.05 and *E*[*R*^(1)^_(1,0,0)_(3)] = 0.65. Case study 2.

**Figure 6.**
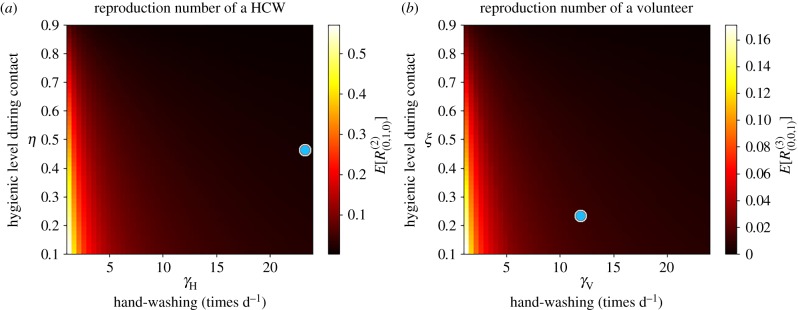
Mean reproduction number of a HCW (*E*[*R*^(2)^_(0,1,0)_], *a*) and a volunteer (*E*[*R*^(3)^_(0,0,1)_], *b*), versus *γ*_H_, *η*, *γ*_*V*_ and *ξ*. Blue dot corresponds to parameter values (*γ*_H_, *η*, *γ*_*V*_, *ξ*) = (24.0, 0.46, 12.0, 0.23) in electronic supplementary material, Table S2, leading to values *E*[*R*^(2)^_(0,1,0)_] = 0.02 and *E*[*R*^(3)^_(0,0,1)_] = 0.01. Case study 2.

For parameter values in the electronic supplementary material, Table S2, we plot in figures 5 and 6 the mean reproduction numbers of the different agents in this ward, for varying values of model parameters. We compute in figure 5 the mean reproduction number of a colonized patient starting the outbreak, among HCWs (*E*[*R*^(1)^_(1,0,0)_(2)]) and volunteers (*E*[*R*^(1)^_(1,0,0)_(3)]), versus (*δ*^−1^_C_, *η*) and (*δ*^−1^_C_, *ξ*), respectively. Our results suggest that transmission from patients to HCWs played a significant role in this outbreak, where a given colonized patient contaminates *E*[*R*^(1)^_(1,0,0)_(2)] = 10.05 HCWs during his/her stay in the ward. On the other hand, our model suggests little transmission from colonized patients to volunteers, with *E*[*R*^(1)^_(1,0,0)_(3)] = 0.65. This remains true even though the low hygienic level during patient–volunteer contacts (*ξ* = 0.23 for volunteers versus *η* = 0.46 for HCWs), and seems to be related to the low intensity of these contacts (*β*_PV_ = 0.2 for volunteers versus *β*_PH_ = 0.72 for HCWs). Stochastic variability of the reproduction numbers *E*[*R*^(1)^_(1,0,0)_(2)] = 10.05 and *E*[*R*^(1)^_(1,0,0)_(3)] = 0.65 can also be assessed by our methodology in §2, in terms of standard deviations SD[*R*^(1)^_(1,0,0)_(2)] = 10.50 and SD[*R*^(1)^_(1,0,0)_(3)] = 0.94. These are readily obtained from the probability distributions computed from equation (2.2).

When looking at possible control strategies, it seems clear that the reproduction number of a colonized patient among HCWs can be significantly reduced by improving the hygienic level of each HCW–patient contact, while reducing the length of stay of each patient does not significantly reduce the infectiousness (i.e. contamination ability) of this patient, and similar comments apply to patient–volunteer contacts.

In figure 6, the mean reproduction number of a contaminated HCW or volunteer is computed for varying values of the hygienic levels during each contact, as well as of the hand-washing rates. The fact that HCWs wash their hands an average of 24 times d^−1^ in this ward keeps the reproduction number of these agents low, and only under significantly low hand-washing compliance levels (*γ*_H_ < 5) a substantial increase for this reproduction number is predicted. Thus, for a particular HCW with low hand-washing compliance level, hygienic level during each HCW–patient contact becomes the most important factor determining the infection spread, and similar comments apply to volunteers.

### 3.3. Assessing environmental contamination

**Figure 7.**
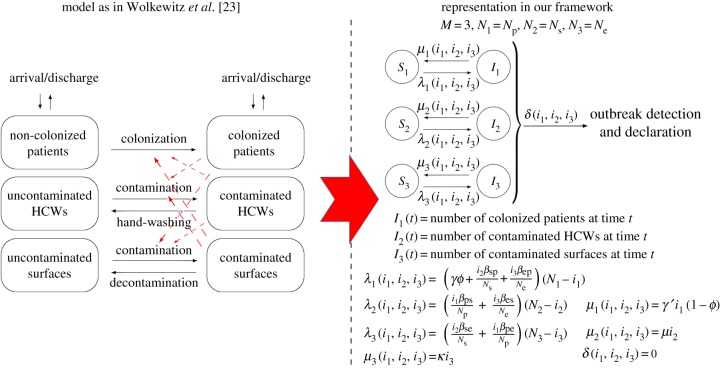
Model by Wolkewitz *et al.* [23] and its corresponding representation in our framework. Our representation leads to the same stochastic process to that in [23]. Case study 3. (Online version in colour.)

The important role played by environmental contamination in nosocomial spread has been discussed in recent works in the field [8,9], since pathogens such as MRSA and *vancomycin-resistant enterococci* (VRE) are able to survive on dry surfaces for weeks [26]. We consider here the model by Wolkewitz *et al.* [23], which incorporates contaminated/non-contaminated surfaces. The authors in [23] consider *N*_p_ patients, *N*_s_ HCWs and *N*_e_ surfaces for analysing an VRE outbreak in the onco-haematological unit at the University Medical Center Freiburg in Germany. Colonized patients are discharged at rate *γ*′, while non-colonized patients are discharged at rate *γ*. Discharged patients are immediately replaced by newly admitted patients, who can be colonized with probability *ϕ*. HCWs wash their hands at rate *μ*, while surfaces are decontaminated at rate *κ*. Transmission between patients, HCWs and surfaces occur at rates (*β*_sp_, *β*_se_, *β*_ps_, *β*_pe_, *β*_es_, *β*_ep_), where s stands for staff (HCWs), p for patients and e for environment (surfaces). In figure 7, we show how this model can be represented into our framework, with the corresponding definition of the function rates. Since no outbreak detection is considered in [23], we set *δ*(*i*_1_, *i*_2_, *i*_3_) = 0.

**Figure 8.**
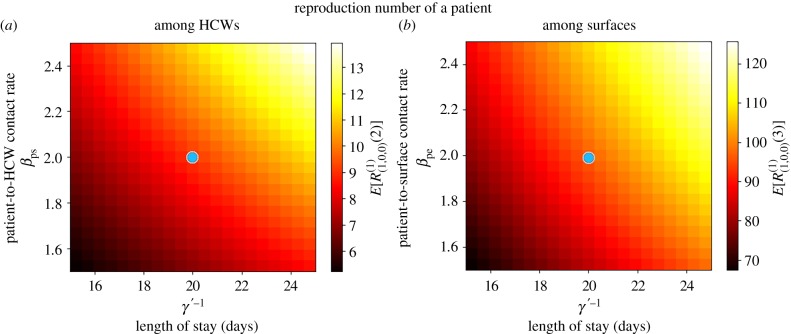
Mean reproduction number of a colonized patient among HCWs (*E*[*R*^(1)^_(1,0,0)_(2)], *a*) and among surfaces (*E*[*R*^(1)^_(1,0,0)_(3)], *b*), versus *γ*′^−1^, *β*_ps_ and *β*_pe_. Blue dot corresponds to parameter values (*β*_ps_, *β*_pe_, *γ*′^−1^) = (2.0, 2.0, 20.0) in electronic supplementary material, Table S3, leading to values *E*[*R*^(1)^_(1,0,0)_(2)] = 9.09 and *E*[*R*^(1)^_(1,0,0)_(3)] = 96.83. Case study 3.

**Figure 9.**
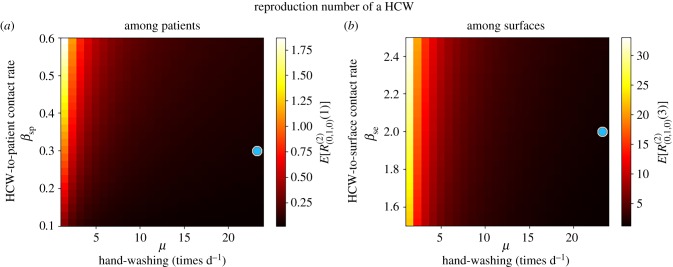
Mean reproduction number of a HCW among patients (*E*[*R*^(2)^_(0,1,0)_(1)], *a*) and among surfaces (*E*[*R*^(2)^_(0,1,0)_(3)], *b*), versus *μ*, *β*_se_ and *β*_sp_. Blue dot corresponds to parameter values (*β*_sp_, *β*_se_, *μ*) = (0.3, 2.0, 24.0) in electronic supplementary material, Table S3, leading to values *E*[*R*^(2)^_(0,1,0)_(1)] = 0.05 and *E*[*R*^(2)^_(0,1,0)_(3)] = 1.64. Case study 3.

**Figure 10.**
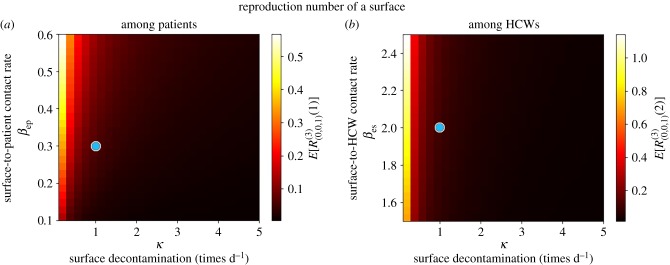
Mean reproduction number of a surface among patients (*E*[*R*^(3)^_(0,0,1)_(1)], *a*) and among HCWs (*E*[*R*^(3)^_(0,0,1)_(2)], *b*), versus *κ*, *β*_es_ and *β*_ep_. Blue dot corresponds to parameter values (*β*_es_, *β*_ep_, *κ*) = (2.0, 0.3, 1.0) in electronic supplementary material, Table S3, leading to values *E*[*R*^(3)^_(0,0,1)_(1)] = 0.06 and *E*[*R*^(3)^_(0,0,1)_(2)] = 0.10. Case study 3.

In figures 8–10, we compute the mean reproduction number of all the agents (i.e. patients, HCWs and surfaces) in this hospital ward, for parameter values in the electronic supplementary material, Table S3, which are the ones considered in [23] for the VRE outbreak in the onco-haematological unit, and carry out a sensitivity analysis for several model parameters. In particular, we plot in figure 8 the mean reproduction number of a colonized patient among HCWs and among surfaces, versus the patient-to-HCW (respectively, patient-to-surface) transmission rate *β*_ps_ (*β*_pe_), and the average length of stay *γ*′^−1^ of any given colonized patient. For the VRE outbreak considered in [23], an average number of *E*[*R*^(1)^_(1,0,0)_(2)] = 9.09 HCWs and *E*[*R*^(1)^_(1,0,0)_(3)] = 96.83 surfaces are contaminated by a colonized patient during his/her stay in the ward, these results suggesting that environmental contamination might be playing a significant role in the infection spread, as suspected by authors in [23]. Stochastic variability of these summary statistics can be represented in terms of the standard deviations SD[*R*^(1)^_(1,0,0)_(2)] = 9.40 and SD[*R*^(1)^_(1,0,0)_(3)] = 73.75, these large quantities suggesting that the corresponding infection processes are highly stochastic. We note that for a colonized patient staying in the ward for an average of 20 days, and an environmental cleaning rate of *κ* = 1 time d^−1^, the same surface can be contaminated several times by this patient during his/her stay. According to results in figure 8, both reducing the average length of stay of patients and decreasing contact rates (i.e. avoiding when possible patient–surface contacts, or improving the hygienic level during each patient–HCW contact) can help to reduce these mean reproduction numbers.

Once a HCW is contaminated, his/her infectious potential can be measured by means of his/her mean reproduction number, which is analysed in figure 9. It seems clear from results in figure 9 that the hand-washing rate *μ* = 24 times d^−1^ allows to keep this mean reproduction number, for a contaminated HCW, low among patients, although it can be still significant (above 1) among surfaces. Results in figure 9 also suggest that HCWs with significantly low hand-hygiene compliance levels (*μ* < 10) could lead to reproduction numbers above 1.75 (among patients) and above 30 (among surfaces), so that our results support the fact that a single HCW with relatively low hand-hygiene compliance level could play a significant infectious role by means of contaminating a large amount of surfaces, and colonizing several patients, until he/she washes his/her hands.

In figure 10, we plot analogous values for a contaminated surface. Although for parameters considered in [23] the reproduction numbers of any given contaminated surface (among HCWs and patients) are relatively low, given the substantial number of surfaces that can be contaminated by a colonized patient (figure 8) or a contaminated HCW with a low hand-hygiene compliance level (figure 9), these numbers should still not be neglected. It seems clear from figure 10 that decontamination rate *κ* = 1 time d^−1^ cannot be considered as optimal during the course of a nosocomial outbreak, since just by increasing this up to *κ* = 2 times d^−1^ a significant reduction in the reproduction number of any contaminated surface could be achieved. This seems to support existing control policies such as the ones recommended within the *national guidelines on the management of outbreaks of norovirus infection in healthcare settings* [27] issued by the National Disease Surveillance Centre in Ireland, which involve cleaning affected areas of the ward twice daily during norovirus outbreaks. Results in figure 10 also suggest that, if *κ* = 1 time d^−1^ had to be maintained for any reason, then recommendations among HCWs and patients on reducing as much as possible *infectious* contacts with surfaces during an outbreak could still have a significant impact in reducing the infectivity of any given contaminated surface, specially among patients.

### 3.4. Incorporating space through room configuration of the ward

**Figure 11.**
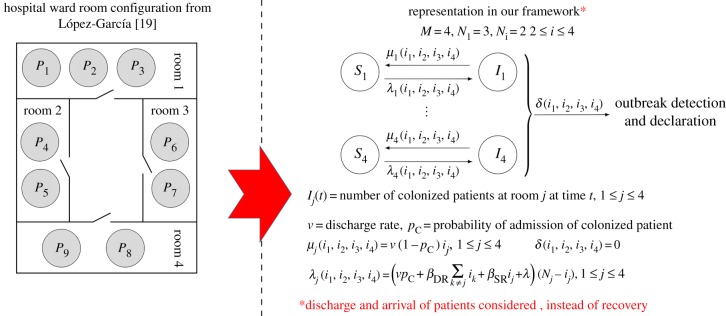
Hospital ward room configuration from López-García [19] and its representation in our framework. Our representation leads to an arguably more realistic stochastic process to that in [19], where patients arrival and discharge are incorporated. Case study 4. (Online version in colour.)

The model by López-García [19] incorporates room configuration into the nosocomial infection dynamics, where the main hypothesis is that for some nosocomial pathogens, the transmission rate between patients in the same room would be higher than the transmission rate for patients in different rooms (this might be the case, for example, when considering airborne transmission [10], if patients in the same room are treated by the same common HCW [15] or when considering isolation rooms where specific control protocols are followed [19]). Since the infection dynamics in [19] are model for an intensive care unit with four rooms, by a simple SIR epidemic model, where no discharge and arrival of patients is considered, we analyse a more realistic scenario here where patients are discharged at rate *ν*, and immediately replaced by newly admitted patients, who can be colonized with probability *p*_C_. A transmission rate *β*_SR_ is considered for patients in the same room, while *β*_DR_ is the transmission rate for patients in different rooms, and HCWs are not explicitly included into the model. A spontaneous colonization rate *λ* is also considered in [19], and no outbreak detection and declaration is assumed so that we set *δ*(*i*_1_, *i*_2_, *i*_3_, *i*_4_) = 0; see figure 11 for the representation into our framework.

**Figure 12.**
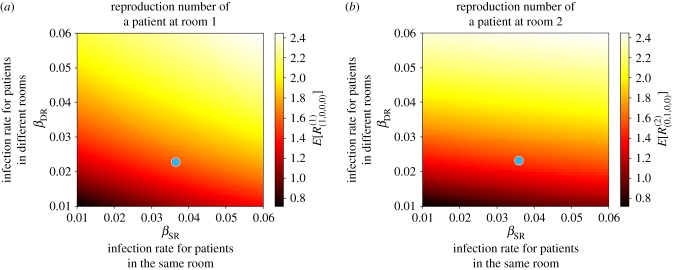
Mean reproduction number of a colonized patient at Room 1 (*E*[*R*^(1)^_(1,0,0,0)_], *a*) and at Room 2 (*E*[*R*^(2)^_(0,1,0,0)_], *b*) starting the outbreak, versus (*β*_SR_, *β*_DR_). Blue dot corresponds to parameter values (*β*_SR_, *β*_DR_) = (0.0366, 0.0238) in electronic supplementary material, Table S4, leading to values *E*[*R*^(1)^_(1,0,0,0)_] = 1.62 and *E*[*R*^(2)^_(0,1,0,0)_] = 1.54. Case study 4.

For parameter values considered in [19], reported in the electronic supplementary material, Table S4, we compute in figure 12 the reproduction number of a colonized patient starting the outbreak at Room 1 (figure 12*a*) and 2 (figure 12*b*), versus transmission rates (*β*_DR_, *β*_SR_). We note that Rooms 3 and 4 are *equivalent* to Room 2, and are thus not analysed. It is interesting to note that for parameter values considered in [19], the reproduction number of a patient at Room 1 is *E*[*R*^(1)^_(1,0,0,0)_] = 1.62, while it is *E*[*R*^(2)^_(0,1,0,0)_] = 1.54 for a patient at Room 2. Stochastic variability of these summary statistics can be represented in terms of the standard deviations SD[*R*^(1)^_(1,0,0,0)_] = 1.73 and SD[*R*^(2)^_(0,1,0,0)_] = 1.67. A threshold behaviour can be observed in both plots in figure 12, where reducing the contact rate between patients in the same room does not seem to have a significant effect on the reproduction number of a patient starting the outbreak at Room 2. For this room, it is the transmission rate between different rooms *β*_DR_ which has a significant impact. This seems to support the idea of implementing patient cohorting as an infection control strategy, where a given HCW treating patients in the same room would avoid, when possible, to treat patients in a different room during the course of a nosocomial outbreak. On the other hand, a parameter threshold can also be observed for a patient starting the outbreak at Room 1, but this threshold depends on a nonlinear combination of the values (*β*_SR_, *β*_DR_). In particular, both reducing the contact rate between patients in the same room and between patients in different rooms can move the value of the reproduction number near or below 1.

### 3.5. Modelling healthcare workers–patient contact network with different healthcare workers infection risk profiles

Finally, we focus here on the model by Temime *et al.* [15], where the potential of some HCWs in a hospital ward to act as super-spreaders during a nosocomial outbreak is assessed. Temime *et al.* [15] consider an hypothetical hospital ward with three types of HCWs: AP1 (a profile involving frequent contacts with a limited number of patients, typically a nurse), AP2 (a profile involving fewer contacts with more patients, typically a physician) and a *peripatetic* HCW (involving a single daily contact with all patients, for instance a therapist or a radiologist). These different HCW profiles lead to different transmission risks, where AP1-patient contacts can be considered as high risk, AP2-patient contacts have moderate risk and peripatetic-patient contacts have low risk [15, fig. 1]. This is encoded here by considering transmission rates *β*_AP1_ > *β*_AP2_ > *β*_Peri_. The authors in [15] consider an hypothetical hospital ward with 18 beds, that all HCWs wash their hands at rate *μ*, and that all patients are discharged at rate *γ*, being immediately replaced by new non-colonized admitted patients. By means of agent-based stochastic simulations, authors simulate the spread of a nosocomial pathogen (using data for MRSA and VRE) in this ward while incorporating details such as the duration of each HCW–patient contact, the probability of pathogen transmission during a 20 min HCW–patient contact, or the existence of day/night HCW shifts.

**Figure 13.**
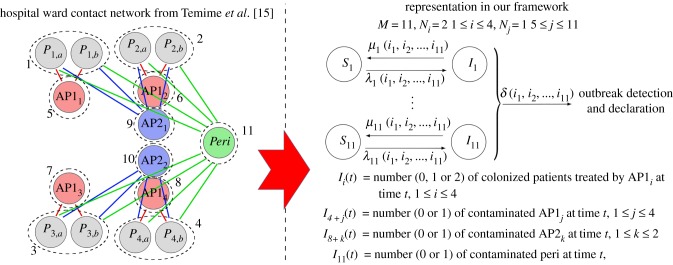
Staff–patient contact network from Temime *et al.* [15] and representation in our framework. Our representation leads to a simplified version of the stochastic process in [15], for a reduced version of the hospital ward represented in [15, fig. 1]. Case study 5. (Online version in colour.)

In figure 13, we represent a simplified version of this model into our framework, for a smaller hospital ward with eight patients, four AP1 HCWs, two AP2 HCWs and one peripatetic HCW, but when considering the same contact network structure than the one studied in [15, fig. 1]. Transmission rates *β*_AP1_, *β*_AP2_ and *β*_Peri_ in electronic supplementary material, Table S5 are obtained by taking into account the duration of each HCW–patient contact type, as well as the probability of pathogen transmission during each contact, by using values in [15, table 1] and following the arguments in [15, supplementary material I]. Since no outbreak detection is considered in [15], we set *δ*(*i*_1_, …, *i*_11_) = 0 and
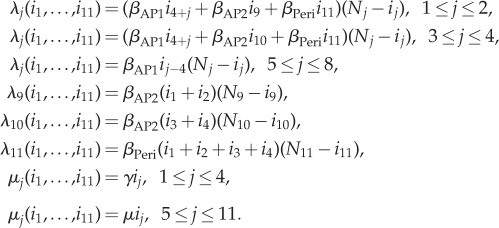

**Figure 14.**
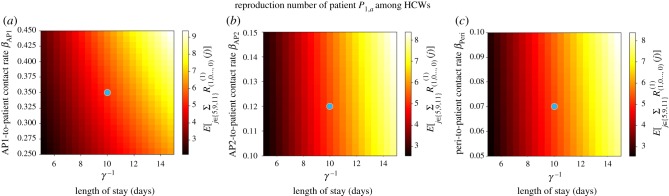
Mean reproduction number of patient *P*_1*a*_ among all HCWs treating him/her (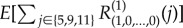), versus *γ*^−1^, *β*_AP1_, *β*_AP2_ and *β*_Peri_, for *μ* = 24 times d^−1^. Blue dot corresponds to parameter values (*β*_AP1_, *β*_AP2_, *β*_Peri_) = (0.35, 0.12, 0.07) in electronic supplementary material, Table S5, leading to value 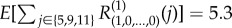. Case study 5.

Given the complexity of this model, we report in table 2 the meanings of our summary statistics in §2. In figure 14, we plot the mean reproduction number of a representative colonized patient (e.g. *P*_1,*a*_) starting the outbreak, among those HCWs that treat him/her (AP1_1_, AP2_1_ and peripatetic). These values are mainly dominated by *β*_AP1_ and *γ*^−1^; that is, by the contact rate for high transmission risk contacts and the length of stay of the patient in the ward. For parameters in the electronic supplementary material, Table S5, a colonized patient contaminates around 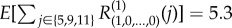 HCWs during his/her stay, with SD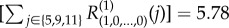. By analysing values of *E*[*R*^(1)^_(1,0, … ,0)_(5)], *E*[*R*^(1)^_(1,0, … ,0)_(9)] and *E*[*R*^(1)^_(1,0, … ,0)_(11)] separately, one can decipher that this corresponds to *E*[*R*^(1)^_(1,0, … ,0)_(5)] = 3.42 contamination events to the AP1_1_, *E*[*R*^(1)^_(1,0, … ,0)_(9)] = 1.19 to the AP2_1_ and *E*[*R*^(1)^_(1,0, … ,0)_(11)] = 0.69 to the peripatetic HCW. However, we note that since AP1_1_ only treats two patients, while the peripatetic treats eight patients, the peripatetic HCW might have his/her hands contaminated for longer periods during a nosocomial outbreak.

**Table 2.** Meaning of our summary statistics for model in figure 13. Case study 5.

*R*^(1)^_(1,0, … ,0)_ = *R*^(1)^_(1,0, … ,0)_(5) + *R*^(1)^_(1,0, … ,0)_(9) + *R*^(1)^_(1,0, … ,0)_(11)	reproduction number of patient *P*_1,*a*_
*R*^(5)^_(0,0,0,0,1,0, … ,0)_ = *R*^(5)^_(0,0,0,0,1,0, … ,0)_(1)	reproduction number of the AP1_1_ HCW
*R*^(9)^_(0, … ,0,1,0,0)_ = *R*^(9)^_(0, … ,0,1,0,0)_(1) + *R*^(9)^_(0, … ,0,1,0,0)_(2)	reproduction number of the AP2_1_ HCW
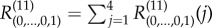	reproduction number of the *peripatetic* HCW

**Figure 15.**
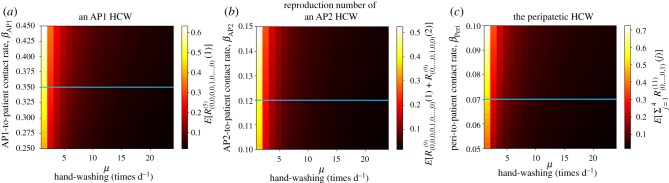
Mean reproduction number of an AP1 (*E*[*R*^(5)^_(0,0,0,0,1,0, … ,0)_(1)], *a*), an AP2 (*E*[*R*^(9)^_(0, … ,0,1,0,0)_(1) + *R*^(9)^_(0, … ,0,1,0,0)_(2)], *b*) and the peripatetic (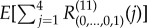, *c*) HCW starting the outbreak, among the patients that they treat, versus *μ*, *β*_AP1_, *β*_AP2_ and *β*_Peri_. Blue line corresponds to parameter values explored in electronic supplementary material, Table S5. Case study 5.

In figure 15, we plot the mean reproduction number of the AP1_1_ (*E*[*R*^(5)^_(0,0,0,0,1,0, … ,0)_(1)]), the AP2_1_ (*E*[*R*^(9)^_(0, … ,0,1,0,0)_(1) + *R*^(9)^_(0, … ,0,1,0,0)_(2)]) and the peripatetic (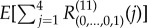) HCW starting the outbreak. Larger values are found for the peripatetic HCW, even though its low transmission risk per contact (*β*_Peri_ < *β*_AP2_ < *β*_AP1_), which is directly related to the large number of patients this peripatetic HCW treats. Larger mean reproduction numbers found for AP1_1_ than for AP2_1_ suggest, however, that there exists a trade-off between the transmission risk profile of each contact (encoded by rates *β*_AP2_ and *β*_AP1_) and the number of patients that each HCW treats (i.e. the particular contact network within the hospital ward). The potential for the peripatetic HCW to act as a super-spreader can be noticed from a combination of results in figures 14 and 15. In particular, we note that the infectious potential of the peripatetic HCW is enhanced by the fact that this HCW might have his/her hands contaminated for long periods, since each of the eight patients treated by this HCW, who might be colonized, contaminates peripatetic HCW hands an average of 0.69 times during their stay. Moreover, it is clear from our results that low hygiene levels during peripatetic-patient contacts (i.e. increasing values of *β*_Peri_) might significantly increase the number of patients that this HCW colonizes until washing his/her hands, and results in figure 15 suggest that the same applies for his/her hand-washing compliance level, which could enhance his/her role as a super-spreader during a nosocomial outbreak.

## 4. Discussion

In this work, we present a unified stochastic modelling framework for the analysis of the spread of nosocomial infections. This unified model allows one to move from more compartment-based models for highly homogeneous scenarios (*M* ≈ 1), to agent-based type models when dealing with highly heterogeneous settings (*M* ≈ *N*, where *N* is the total number of individuals in the population). We note that when considering the asymptotic case *M* = *N*, with *N*_*j*_ = 1 for all 1 ≤ *j* ≤ *M*, the resulting space of states 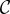 contains 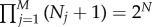 states, as in this case one is in fact analysing the SIS epidemic model on a network [18,19]. Our unified framework allows one to consider different hypotheses related to the detection and declaration of the nosocomial outbreak, or to analyse the long-term infection spread when this detection is not relevant. This versatile model also allows us to represent a wide range of agents involved in the nosocomial outbreak, to account for hand-washing compliance levels, environmental cleaning, patients arrival/discharge, spatial components such as the hospital ward room configuration, different types of HCWs corresponding to different pathogen transmission risks, as well as specific patient–staff contact network topologies.

Our methodology within this unified framework allows one to exactly analyse the probability distribution of the exact reproduction number of each agent in the ward. Moreover, this summary statistic can be split into several ones accounting for the infections caused by a given individual among individuals of a particular type. This translates into analysing the infectiousness of patients, HCWs, volunteers or surfaces among individuals of each of these groups, so that the role played by each potential contact transmission route can be assessed for nosocomial outbreaks corresponding to different healthcare facilities and pathogens. To the best of our knowledge, this is the first time that this analytical approach, which has been usually neglected when analysing infection spread among individuals in populations of moderate-to-large sizes—due to computational constraints—is applied in the area of nosocomial infections where populations are usually small and heterogeneous, making its implementation feasible. We note that, although the focus here has been on studying the reproduction number of each individual, alternative summary statistics of interest allowing for first-step analysis (such as the length or the final size of the outbreak [18,19]) could be analysed in the same way by means of this unified framework and our methodology in §2.

Our unified framework, together with the analytical approach in §2, allows one to exactly compute the corresponding reproduction numbers and to use these to assess the role played by the different routes of infection during a nosocomial outbreak. At the same time, the fact that all scenarios in §3—and potentially others—can be represented into our unified framework, means that computer codes developed for solving equations (2.2)–(2.3) for the general model in figure 1 can be readily applied in all these scenarios, just by specifying the corresponding *μ*_*j*_(*i*_1_, …, *i*_*M*_), *λ*_*j*_(*i*_1_, …, *i*_*M*_) and *δ*(*i*_1_, …, *i*_*M*_) rates. On the other hand, we acknowledge that this unified stochastic framework represented by the diagram in figure 1 entails several simplifying assumptions and limitations. The constant size assumed for each compartmental level means that the total number of agents of each type (patients, HCWs, surfaces, volunteers, etc.) remains constant during the course of the nosocomial outbreak. When focusing on patients, this is only appropriate under high demand situations, where the time during which any given bed is empty is short enough and can be neglected in the corresponding model. Under moderate demand, and if one needs to incorporate empty beds explicitly in the model, the stochastic process in §2 could be modified so that *S*_1_(*t*) (if *j* = 1 represents the compartmental level corresponding to patients) is incorporated as an additional variable into the continuous-time Markov chain 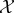, so that *S*_1_(*t*) + *I*_1_(*t*) is not necessarily constant throughout time. Moreover, more complex situations such as nosocomial outbreaks occurring across several hospital wards, with patient movement between wards, or competitive scenarios where several bacterial strains (e.g. antibiotic-sensitive versus antibiotic-susceptible [28]) are spreading simultaneously within the same hospital ward, cannot be directly represented into our framework by just specifying rates *μ*_*j*_(*i*_1_, …, *i*_*M*_), *λ*_*j*_(*i*_1_, …, *i*_*M*_) and *δ*(*i*_1_, …, *i*_*M*_). Instead, alternative diagramatic representations to that in figure 1 should be explored, potentially including movement of agents between different compartmental levels.

We also note that our methodology directly relies on the fact that the model proposed is a continuous-time Markov chain, so that events are Markovian and inter-event times are assumed to be exponentially distributed. While this is a typical assumption in the literature when analysing nosocomial outbreaks from a stochastic perspective, we acknowledge that the exponential distribution might not be appropriate for some particular events in these processes, such as patients' lengths of stay. Although relaxing the Markovian assumption in these models is out of the scope of this paper, it is worth to point out here that some attempts have already been made in this area, some of them based on the use of phase-type distributions for incorporating these non-Markovian events [29,30].

Finally, we acknowledge here that additional limitations of our approach are of computational nature, related to solving systems of around 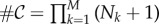 linear equations. However, populations usually involved in nosocomial outbreaks are small enough for this methodology to be efficiently implemented, where specific procedures for dealing with systems of equations involving highly sparse matrices can be specially useful. We also note that while *N* = 20 + 5 + 100 = 125 individuals in case study 3 (patients, HCWs and surfaces) lead to analysing a stochastic process with 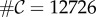 states, only *N* = 2 + 2 + 2 + 2 + 1 + 1 + 1 + 1 + 1 + 1 + 1 = 15 individuals in case study 5 (patients, AP1, AP2 and peripatetic HCWs) lead to 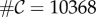 states, which is directly related to the high level of individual heterogeneity introduced into this model (encoded by the number of compartmental levels *M* = 3 versus *M* = 11). These comments suggest that while agent-based simulation approaches should prevail under highly heterogeneous scenarios, such as the complete model by Temime *et al.* [15], more homogeneous or low-to-moderate heterogeneous settings allow for this exact approach to be implemented.

## Supplementary Material

Supplementary Material

## Data accessibility

Computer codes (in *Python*) to reproduce our results in §3 are available in [31].
