# Supplementary material for: A unified stochastic modelling framework for the spread of nosocomial infections
Source: J R Soc Interface. 2018 Jun 13;15(143):20180060. doi: 10.1098/rsif.2018.0060 (PMC6030628; doi:10.1098/rsif.2018.0060)
Supplement: Supplementary Material [file rsif20180060supp1.pdf]

Supplementary material: *A unified stochastic modelling framework for the spread of nosocomial infections*

Martín López-García[1*], Theodore Kypraios[2]

[1]*School of Mathematics, University of Leeds, LS2 9JT Leeds, UK*
[2]*School of Mathematical Sciences, University of Nottingham, NG7 2RD Nottingham, UK*

Abstract

In this Supplementary Material, we discuss about how systems of equations in Ref. [1] can be represented in matrix form and iteratively solved. Moreover, we report here parameter values considered in case studies 1-5 in Ref. [1], obtained from Refs. [2, 3, 4, 5, 6], and summarise in Table S6 the function rates $\lambda_j(i_1,\ldots,i_M)$, $\mu_j(i_1,\ldots,i_M)$ and $\delta(i_1,\ldots,i_M)$ for these case studies.

1 Matrix-oriented solutions

1.1 Number of infections caused by an individual at compartment j until he/she is removed or the outbreak is detected

The objective here is to compute probabilities

$$\nu^{(j)}_{(i_1,\ldots,i_M)}(n) \quad = \quad \mathbb{P}(R^{(j)}_{(i_1,\ldots,i_M)} = n), \quad n \geq 0,$$

by solving the systems of equations given by [1, Eq. (2.3)]. We can rewrite this system into a matrix equation of the form

$$\mathbf{D}^{(j)}\boldsymbol{\nu}^{(j)}(n) \quad = \quad \mathbf{e}^{(j)}(n), \qquad (1)$$

where matrix $\mathbf{D}^{(j)}$ is independent of the value $n \geq 0$, while column vectors $\boldsymbol{\nu}^{(j)}(n)$ and $\mathbf{e}^{(j)}(n)$ depend on this value. In particular,

$$(\mathbf{D}^{(j)})_{(i_1,\ldots,i_M),(i_1,\ldots,i_k-1,\ldots,i_M)} = \frac{1}{\theta_{(i_1,\ldots,i_M)}}\mu_k\left(i_k 1_{k\neq j}\right.$$
$$\left. +(i_k-1)1_{k=j}\right), \quad 1 \leq k \leq M,$$
$$(\mathbf{D}^{(j)})_{(i_1,\ldots,i_M),(i_1,\ldots,i_k+1,\ldots,i_M)} = \frac{1}{\theta_{(i_1,\ldots,i_M)}}\Bigg(\lambda_k$$
$$+\sum_{l=1,\ l\neq j}^{M}\lambda_{lk}i_l + (i_j-1)\lambda_{jk}\Bigg)(N_k - i_k), \quad 1 \leq k \leq M,$$
$$(\mathbf{e}^{(j)}(n))_{(i_1,\ldots,i_M)} = \frac{1}{\theta_{(i_1,\ldots,i_M)}}\Bigg(1_{n>0}\sum_{k=1}^{M}(N_k-i_k)\lambda_{jk}$$
$$\times\,\nu^{(j)}_{(i_1,\ldots,i_k+1,\ldots,i_M)}(n-1) + 1_{n=0}(\mu_j + \delta(i_1,\ldots,i_M))\Bigg).$$

Dimensionality of system in Eq. (1) is not $\#\mathcal{C} = \prod_{k=1}^{M}(N_k+1)$, but $N_j\prod_{k=1,\ k\neq j}^{M}(N_k+1)$, since probabilities $\nu^{(j)}_{(i_1,\ldots,i_M)}(n)$ are only defined for states $(i_1,\ldots,i_M) \in \mathcal{C}$ with $i_j > 0$. Moreover, we are storing in Eq. (1) probabilities $\nu^{(j)}_{(i_1,\ldots,i_M)}(n)$ in the column vector

$$\boldsymbol{\nu}^{(j)}(n) \quad = \quad \begin{pmatrix} \nu^{(j)}_{(0,0,\ldots,0,0)}(n) \\ \nu^{(j)}_{(0,0,\ldots,0,1)}(n) \\ \nu^{(j)}_{(0,0,\ldots,0,2)}(n) \\ \vdots \\ \nu^{(j)}_{(0,0,\ldots,0,N_M)}(n) \\ \nu^{(j)}_{(0,0,\ldots,1,0)}(n) \\ \nu^{(j)}_{(0,0,\ldots,1,1)}(n) \\ \nu^{(j)}_{(0,0,\ldots,1,2)}(n) \\ \vdots \\ \nu^{(j)}_{(N_1,N_2,\ldots,N_{M-1},N_M)}(n) \end{pmatrix},$$

so that each row in this matrix system represents a state $(i_1,\ldots,i_M) \in \mathcal{C}$, with $i_j > 0$. Due to the lexicographic order followed above when ordering these states by rows, each state $(i_1,\ldots,i_M)$ with $i_j > 0$ corresponds to the $\sum_{k=1}^{M}(1_{k\neq j}i_k + 1_{k=j}(i_j-1))\prod_{p=k+1}^{M}(1_{p\neq j}(N_p+1) + 1_{p=j}N_p)^{th}$ row (*i.e.,* equation) in Eq. (1). Finally, since matrix $\mathbf{D}^{(j)}$ is significantly sparse, for numerical results in [1, Section 3] we solve this system of linear equations by using the *scipy.sparse.linalg* Python package. This involves solving Eq. (1) for $n = 0$, and then iteratively solving it for values $n \geq 1$ using probabilities $\nu^{(j)}_{(i_1,\ldots,i_M)}(n-1)$, which are stored in column vector $\boldsymbol{\nu}^{(j)}(n-1)$ previously computed.

*Author for correspondence (m.lopezgarcia@leeds.ac.uk)

1.2 Number of infections caused by an individual at compartment j, among individuals at compartment k, until he/she is removed or the outbreak is detected

The objective here is to compute probabilities

$$\nu^{(j)}_{(i_1,\dots,i_M)}(k;n) \;=\; \mathbb{P}(R^{(j)}_{(i_1,\dots,i_M)}(k)=n), \quad n\geq 0,$$

by solving the systems of equations given by [1, Eq. (2.2)]. Again, we can construct and iteratively solve matrix systems of the form

$$\mathbf{D}^{(j)}(k)\boldsymbol{\nu}^{(j)}(k;n) \;=\; \mathbf{e}^{(j)}(k;n),$$

where

$$(\mathbf{D}^{(j)}(k))_{(i_1,\dots,i_M),(i_1,\dots,i_l-1,\dots,i_M)} = \frac{1}{\theta_{(i_1,\dots,i_M)}}\mu_l\,(i_l 1_{l\neq j} + (i_l-1)1_{l=j}), \quad 1\leq l\leq M,$$

$$(\mathbf{D}^{(j)}(k))_{(i_1,\dots,i_M),(i_1,\dots,i_l+1,\dots,i_M)} = \frac{1}{\theta_{(i_1,\dots,i_M)}}\Bigg(1_{l\neq k}\,(\lambda_l + \sum_{p=1}^{M}\lambda_{pl}i_p)(N_l-i_l) + 1_{l=k}(\lambda_l + \sum_{p=1,\ p\neq j}^{M}\lambda_{pl}i_p + \lambda_{jl}(i_j-1))\,(N_l-i_l)\Bigg), \quad 1\leq l\leq M,$$

$$(\mathbf{e}^{(j)}(k;n))_{(i_1,\dots,i_M)} = \frac{1}{\theta_{(i_1,\dots,i_M)}}\Bigg(1_{n>0}(N_k-i_k)\lambda_{jk} \times \nu^{(j)}_{(i_1,\dots,i_k+1,\dots,i_M)}(k;n-1) + 1_{n=0}(\mu_j+\delta(i_1,\dots,i_M))\Bigg),$$

and where probabilities $\nu^{(j)}_{(i_1,\dots,i_M)}(k;n)$ are stored in the column vectors $\boldsymbol{\nu}^{(j)}(k;n)$, as in subsection 1.1 of this Supplementary Material.

2 Parameter values for case studies 1-5

In Tables S1-S5, we report parameter values considered in case studies 1-5 in Ref. [1], directly obtained from Refs. [2, 3, 4, 5, 6]. In Table S6, we summarise the functional forms of rates $\lambda_j(i_1,\dots,i_M)$, $\mu_j(i_1,\dots,i_M)$ and $\delta(i_1,\dots,i_M)$ for case studies 1-5, according to the corresponding model assumptions and model parameters described in Ref. [1].

	Meaning	Value
N_p	Number of patients	20
N_{HCW}	Number of HCWs	3
μ	Patient discharge rate	0.1
γ	Patient detection rate	0.1
μ'	HCW hand-washing rate	14
β	HCW-to-patient colonization rate	$\frac{1}{6}$
β'	Patient-to-HCW contamination rate	$\frac{1}{6}$
σ	Fraction of admitted patients colonized	0.01

Table S1: Parameter values from Artalejo (2014) [2], for the spread of MRSA in an hypothetical intensive care unit. Time units: *days*. Case study 1

	Meaning	RICU
N_P	Number of patients	7
N_{HCW}	Number of HCWs	14
N_V	Number of volunteers	2
φ	Fraction of admitted patients colonized	0.165
$\frac{1}{\delta_U}$	Length of stay, non-colonized patients	7
$\frac{1}{\delta_C}$	Length of stay, colonized patients	13
η	Hygienic level, HCW-patient	0.46
ξ	Hygienic level, volunteer-patient	0.23
β_{PH}	Patient-HCW contact rate	0.72
β_{PV}	Patient-volunteer contact rate	0.20
γ_H	HCW hand-washing rate	24
γ_V	Volunteer hand-washing rate	12

Table S2: Parameter values from Wang et al. (2011) [3], for the spread of MRSA in the Respiratory Intensive Care Unit (RICU) at Beijing Tongren Hospital. Time units: *days*. Case study 2

	Meaning	Value
N_p	Number of patients	20
N_s	Number of HCWs	5
N_e	Number of surfaces	100
ϕ	Fraction of admitted patients colonized	0.1
γ	Discharge rate, non-colonized patients	0.1
γ'	Discharge rate, colonized patients	0.05
μ	Staff decontamination rate	24
κ	Surfaces decontamination rate	1
β_{sp}	Staff-to-patient colonization rate	0.3
β_{se}	Staff-to-surface contamination rate	2
β_{ps}	Patient-to-staff contamination rate	2
β_{pe}	Patient-to-surface contamination rate	2
β_{es}	Surface-to-staff contamination rate	2
β_{ep}	Surface-to-patient colonization rate	0.3

Table S3: Parameter values from Wolkewitz et al. (2008) [4], for an VRE outbreak in the onco-haematological unit at the University Medical Center Freiburg in Germany. Time units: *days*. Case study 3

References

[1] López-García M, Kypraios T (2018) *A unified stochastic modelling framework for the spread of nosocomial infections.* Journal of the Royal Society Interface 20180060. http://dx.doi.org/10.1098/rsif.2018.0060.

[2] Artalejo, JR (2014) *On the Markovian approach for modeling the dynamics of nosocomial infections.* Acta Biotheoretica, 62: 15-34.

[3] Wang J, Wang L, Magal P, Wang Y, Zhuo J, Lu X, Ruan S (2011) *Modelling the transmission dynamics of meticillin-resistant Staphylococcus aureus in Beijing Tongren hospital.* Journal of Hospital Infection, 79: 302-308.

[4] Wolkewitz M, Dettenkofer M, Bertz H, Schumacher

		Rate function			
CS	M	$\mu_j(i_1,\dots,i_M) = \mu_j i_j$	$\lambda_j(i_1,\dots,i_M) = (N_j - i_j)\left(\lambda_j + \sum_{k=1}^{M} \lambda_{kj} i_k\right)$		$\delta(i_1,\dots,i_M)$
1	2	$\mu_1 = (1-\sigma)\mu$, $\mu_2 = \mu'$	$\lambda_1 = \sigma\mu$, $\lambda_2 = 0$	$\lambda_{12} = \beta'$, $\lambda_{21} = \beta$	$\delta(i_1, i_2) = \gamma i_1$
2	3	$\mu_1 = \delta_C(1-\varphi)$, $\mu_2 = \gamma_H$<br>$\mu_3 = \gamma_V$	$\lambda_1 = \delta_U\varphi$, $\lambda_2 = 0$<br>$\lambda_3 = 0$	$\lambda_{12} = \frac{1-\eta}{N_P}\beta_{PH}$, $\lambda_{13} = \frac{1-\xi}{N_P}\beta_{PV}$<br>$\lambda_{21} = \frac{1-\eta}{N_P}\beta_{PH}$, $\lambda_{23} = 0$<br>$\lambda_{31} = \frac{1-\xi}{N_P}\beta_{PV}$, $\lambda_{32} = 0$	$\delta(i_1, i_2, i_3) = 0$
3	3	$\mu_1 = \gamma'(1-\phi)$, $\mu_2 = \mu$<br>$\mu_3 = \kappa$	$\lambda_1 = \gamma\phi$, $\lambda_2 = 0$<br>$\lambda_3 = 0$	$\lambda_{12} = \frac{\beta_{ps}}{N_p}$, $\lambda_{13} = \frac{\beta_{pe}}{N_p}$<br>$\lambda_{21} = \frac{\beta_{sp}}{N_s}$, $\lambda_{23} = \frac{\beta_{se}}{N_s}$<br>$\lambda_{31} = \frac{\beta_{ep}}{N_e}$, $\lambda_{32} = \frac{\beta_{es}}{N_e}$	$\delta(i_1, i_2, i_3) = 0$
4	4	$\mu_j = \nu(1-p_C)$, $1 \le j \le 4$	$\lambda_j = \nu p_C + \lambda$, $1 \le j \le 4$	$\lambda_{jk} = \beta_{DR}$, $1 \le j \ne k \le 4$<br>$\lambda_{jj} = \beta_{SR}$, $1 \le j \le 4$	$\delta(i_1,\dots,i_4) = 0$
5	11	$\mu_j = \gamma$, $1 \le j \le 4$<br>$\mu_j = \mu$, $5 \le j \le 11$	$\lambda_j = 0$, $1 \le j \le 11$	$\lambda_{51} = \lambda_{15} = \lambda_{62} = \lambda_{26} = \beta_{AP1}$<br>$\lambda_{73} = \lambda_{37} = \lambda_{84} = \lambda_{48} = \beta_{AP1}$<br>$\lambda_{91} = \lambda_{19} = \lambda_{92} = \lambda_{29} = \beta_{AP2}$<br>$\lambda_{10,3} = \lambda_{3,10} = \lambda_{10,4} = \lambda_{4,10} = \beta_{AP2}$<br>$\lambda_{11,1} = \lambda_{1,11} = \lambda_{11,2} = \lambda_{2,11} = \beta_{Peri}$<br>$\lambda_{11,3} = \lambda_{3,11} = \lambda_{11,4} = \lambda_{4,11} = \beta_{Peri}$<br>For others (j,k): $\lambda_{jk} = 0$	$\delta(i_1,\dots,i_{11}) = 0$

Table S6: Functional forms for case studies 1-5 (CS 1-5)

	Meaning	Value
N_p	Number of patients	9
ν	Discharge rate	0.1
p_C	Fraction of admitted patients colonized	0.01
β_{DR}	Cross-colonization rate, different rooms	0.0238
β_{SR}	Cross-colonization rate, same room	0.0366
λ	Spontaneous colonization rate	0.0037

Table S4: Parameter values from López-García (2016) [5], for an MRSA outbreak in an intensive care unit with four rooms. Parameter values ν and p_C from Artalejo et al. (2014) [2]. Time units: *days*. Case study 4

	Meaning	Value
β_{AP1}	Patient-AP1 transmission rate	0.35
β_{AP2}	Patient-AP2 transmission rate	0.12
β_{Peri}	Patient-peripatetic transmission rate	0.07
μ	Hand-washing rate for all HCWs	$1-24$
γ^{-1}	Length of stay for all patients	10

Table S5: Parameter values from Temime et al. (2009) [6], for a bacterial outbreak in an hypothetical intensive-care unit. Time units: *days*. Case study 5

M, Huebner J (2008) *Environmental contamination as an important route for the transmission of the hospital pathogen VRE: modelling and prediction of classical interventions.* Infectious Diseases: Research and Treatment, 1.

[5] López-García M (2016) *Stochastic descriptors in an SIR epidemic model for heterogeneous individuals in small networks.* Mathematical Biosciences, 271: 42-61.

[6] Temime L, Opatowski L, Pannet Y, Brun-Buisson C, Boëlle PY, Guillemot D (2009) *Peripatetic health-care workers as potential superspreaders.* Proceedings of the National Academy of Sciences, 106: 18420-18425.
